# Structural equation and log-linear modeling: a comparison of methods in the analysis of a study on caregivers' health

**DOI:** 10.1186/1471-2288-6-49

**Published:** 2006-10-12

**Authors:** Bin Zhu, Stephen D Walter, Peter L Rosenbaum, Dianne J Russell, Parminder Raina

**Affiliations:** 1 Centre for Clinical Epidemiology and Community Studies, Sir Mortimer B. Davis-Jewish General Hospital, Montreal, Quebec, Canada; 2 Department of Clinical Epidemiology & Biostatistics, Faculty of Health Sciences, McMaster University, Hamilton, Ontario, Canada; 3 McMaster University Evidenced-based Practice Centre, Faculty of Health Sciences, McMaster University, Hamilton, Ontario, Canada; 4 Department of Paediatrics, Faculty of Health Sciences, McMaster University, Hamilton, Ontario, Canada; 5 *CanChild *Centre for Childhood Disability Research, McMaster University, Hamilton, Ontario, Canada; 6 School of Rehabilitation Science, McMaster University, Hamilton, Ontario, Canada

## Abstract

**Background:**

In this paper we compare the results in an analysis of determinants of caregivers' health derived from two approaches, a structural equation model and a log-linear model, using the same data set.

**Methods:**

The data were collected from a cross-sectional population-based sample of 468 families in Ontario, Canada who had a child with cerebral palsy (CP). The self-completed questionnaires and the home-based interviews used in this study included scales reflecting socio-economic status, child and caregiver characteristics, and the physical and psychological well-being of the caregivers. Both analytic models were used to evaluate the relationships between child behaviour, caregiving demands, coping factors, and the well-being of primary caregivers of children with CP.

**Results:**

The results were compared, together with an assessment of the positive and negative aspects of each approach, including their practical and conceptual implications.

**Conclusion:**

No important differences were found in the substantive conclusions of the two analyses. The broad confirmation of the Structural Equation Modeling (SEM) results by the Log-linear Modeling (LLM) provided some reassurance that the SEM had been adequately specified, and that it broadly fitted the data.

## Background

The use of SEM analysis has increased in recent years, especially in social science, education, business, medicine and biological science [1]. The capacity of SEM to distinguish between indirect and direct relationships between variables and to specify structural relations among latent variables differentiates SEM from other simpler modeling processes. Also, the flexibility of SEM allows the researcher to model data structures which violate traditional model assumptions, such as heterogeneous error variances and correlated errors. However, the application of SEM models is often complex in practice, and it requires both theory and data considerations to drive the decision-making in its development and validation. Judgement is required throughout the process, and a strong background in the content area and the causal hypothesis framework by the investigators is important. Particularly controversial areas are the testing of model fit and the iterative model re-specification. For example, non-convergence of parameter estimation is not uncommon because often there are many parameters and relatively limited sample sizes. Sample size should not be small as SEM relies on tests which are sensitive to sample size as well as to the magnitude of differences in covariance matrices. Compared to regression and factor analysis, the SEM is a relatively young field; with its roots in papers that appeared only in the late 1960s, the methodology has limitations and is still considered to be "under construction" [2].

In a recent study [3], we used the SEM approach to examine the causal relationships among the factors relevant to the physical and psychological health of caregivers of children with cerebral palsy. The details of these theoretical frameworks and the results of the SEM analyses are described in detail elsewhere [3,4].

In our SEM analysis of the caregiver study [3], there had been concern initially about the possible instability in the SEM results, because of the large number of parameters to be estimated for the given amount of data available. At the outset, our conceptual SEM model involved 121 parameters (the initial measurement model included three types of parameters to be estimated: variances for exogenous variables, covariances between latent factors, and factor loadings), with approximately 33 indicators (there were at least 3 indicators per latent construct, and we hypothesized 11 latent constructs). Although the available sample size of 486 was quite large, we were concerned that it might nevertheless be inadequate for the estimation of so many parameters. Guidelines in this area suggest a minimum of 5 observations per parameter are needed [5]. Another rule of thumb, based on Stevens [6], is to have at least 15 cases per measured variable or indicator. The researcher should go beyond these minimum sample size recommendations when data are non-normal (e.g. skewed or kurtotic) or incomplete, so it was debatable whether this standard had been met in our analysis.

Accordingly, a LLM analysis was carried out as an adjunct to the SEM analysis. The LLM method is appropriate for multivariate data arranged in contingency table format. One advantage of the LLM approach is that once the variables have been categorized, there are no further distributional requirements to be met. Another advantage is that the LLM should have greater stability, because its factors have fewer levels than those in SEM, and also the number of factors might be reduced in LLM. A disadvantage arises because of the loss of information in the categorical data, as opposed to their original continuous form; hence the explanatory power of the LLM may also be reduced.

The objective of the present paper was to reanalyse the data from the Caregiver study using a LLM method and see whether the results were comparable to those from the SEM. To the extent that similar conclusions emerge, one would have greater confidence in the results from the more complex, and assumption-dependent SEM. If the results of the SEM and LLM turn out to be substantially different, we would take those observations as a signal of possible over-fitting of the data by the more complex SEM, whose results would then be much less trustworthy. In such a situation, one might prefer to report the findings using the LLM, with accordingly less emphasis on more subtle features of the data (e.g. indirect associations), and less attempt to draw causal conclusions.

## Methods

### Data

The Caring about Caregiver study drew on a population from a previous study, the Ontario Motor Growth (OMG) study [7], which had included 657 families, of which 632 families were still available [for further study]. Of these, 570 were contactable and eligible to participate in the Caregiver study, of whom 503 (88.24%) consented and 468 (82.10%) were interviewed. The data collection process consisted of two steps: a self-report, mailed questionnaire followed by a home-based interview with the primary caregiver of the child with CP. A cross-sectional design was used to collect information about socio-economic variables, child characteristics, caregiving demands, measures of self-perception and coping factors, as well as information about the physical and psychological wellbeing of caregivers. The primary caregiver in each family was defined as the person who is most responsible for the day-to-day decision-making and care of the child. Only one primary caregiver per household was self-selected for this study. The details of data collection and the characteristic of this sample were described elsewhere [3].

**Figure 1:**
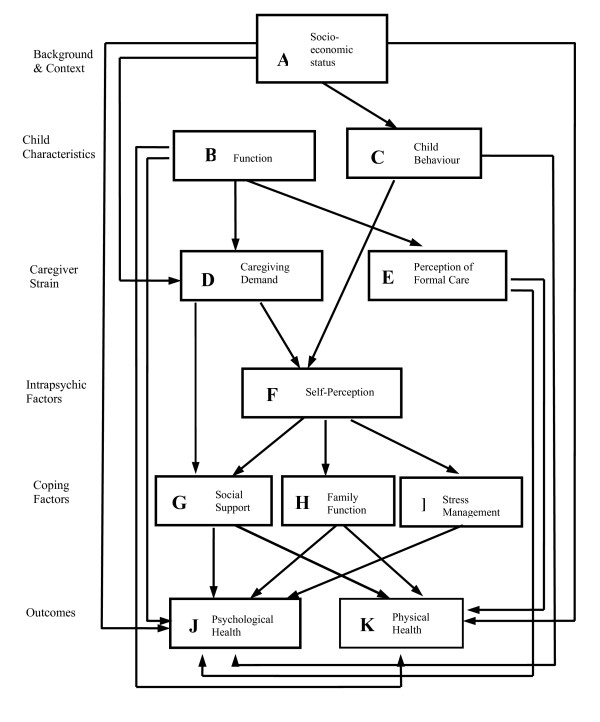
Conceptual model of the caregiving process among caregivers of a pediatric population.

The SEM study proposed a comprehensive model of factors affecting the health of caregivers of children with disabilities (Figure 1). The relevant factors included Background and Context, Child Characteristics, Caregiver Strain, Intrapsychic Factors, Coping Factors and Health Outcomes. The observed variables from each of these domains were chosen to represent the relevant constructs in the SEM methodology. A list of the observed variables and latent variables used in the SEM is presented in Table 1.

**Table 1:** Descriptions of variables

Constructs	Indicators	Measures	Description of Measure
Psychological health: the mental and emotional health of the caregiver	Chronicity of distress Distress Scaled score of role emotional Scaled score of mental health Reported health transitions	National Population Health Survey (NPHS): MH Q1A to Q1F [33] NPHS: MH Q1G-Q1L[33] Medical Outcomes Study: Short Form 36 Health Survey (SF36) [34]	Based on a subset of questions related to mental and emotional health from the Composite International Diagnostic Interview (CIDI). The SF36 is a generic measure of health concepts related to everyone's functional status and well-being.
Physical health: the subjective and objective measures of the physical health of the caregiver	Scaled score of general health Scaled score of physical functioning Scaled score of role-physical Scaled score of bodily pain Scaled score of vitality Presence of chronic conditions (caregiver)	Medical Outcomes Study: Short Form 36 Health Survey (SF36)[34] National Longitudinal Study of Children and Youth (NLSCY): CHRON Q1 [35]	The SF36 is a generic measure of health concepts related to everyone's functional status and well-being. Caregiver responds yes/no to a list of chronic health conditions defined as lasting or expected to last 6 months or more and diagnosed by a health care professional.
Social support: the social relationships that the caregiver has with family, friends and others.	Social provision scale Existence of possible persons to be contacted Number of contacts for all categories Social Functioning from SF36	Social Provision Scale NLSCY: SUP-Q1A to SUP-Q2D [35] Social Network & Frequency of Contact Index: NPHS: SUP-Q7A to SUP-Q7H [33] SF36 [34]	A short version of the Social Provisions Scale by Cutrona ussell, which measures perceived social support from family and friends. Social Network summarizes the existence of possible persons in the caregiver's social network. Contact Index measures the number of contacts the caregiver has had with family, friends and neighbours in the past 12 months. SF36
Family functioning: the level of family functioning and cohesion	Family functioning (caregiver)	Family Assessment Device (FAD) in NLSCY (FNC-Q1A to Q1M) [35]	A global assessment of family functioning and an indication of the quality of relationships between parents or partners.
Self-perception: elements of self-concept and control affecting the caregivers live	Mastery (caregiver) Self esteem (caregiver)	NPHS: MAST-Q1 [36] NPHS: ESTEEM Q1 [36]	A scale that measures elements of self-concept and control affecting the caregiver's lives. A scale of six items that measure caregiver self-esteem.
Stress management: the behaviours and practices of the caregiver in response to life problems and situations.	Integration, cooperation, optimism Support, esteem, stability Medical communication & consultation Mastery scale summary score Self esteem summary score	Coping Health Inventory for Parents (CHIP) [37]	Assesses caregivers' appraisal of their coping responses to the management of family life when they have a child member who is seriously and/or chronically ill.
Perception of formal care: caregiver strain	Enabling and partnership Providing general information Providing specific information Coordinating care Respectful and supportive	Measures of Processes of Care (MPOC) [38]	Measures the caregiver's perceptions of the extent to which specific behaviors of health professionals occur.
Caregiving demands: cross-pressures and dilemmas related to caregiving and occupation.	Caregiving assistance performed by primary caregiver in self care Caregiver assistance in mobility	Pediatric Evaluation of Disability Inventory (PEDI) Parts II and III Caregiver Assistance (Self-Care and Mobility) [39]	Measures the typical amount of caregiver assistance provided to the child during the completion of basic functional activities in areas of self-care and mobility
Child disability: child's level of motor severity and cognitive function, medical problems, and extent of independence in daily activities.	Functional Self Care Functional Mobility	Pediatric Evaluation of Disability Inventory (PEDI) Part 1: Functional Skills (Self-Care and Mobility Domains) [39]	Child is rated as capable/unable on a list of items (73 self care items and 54 mobility items)
Child behaviour: child's feelings, behaviour and social function	Conduct disorder Hyperactivity Emotional disorder	Survey Diagnostic Instrument (SDI) [40]	The SDI is a subset of 24 items of the Child Behaviour Checklist that breaks down into three scales: conduct disorder, hyperactivity and emotional disorder
Socio-economic status: social and economic characteristics of the family	Education Level Gross household income	NLSCY: EDUC-Q2, EDUC-Q3 [35] NLSCY: INCOM-Q3B [35]	Questions related to highest level of education caregiver completed. Question about the annual gross household income

### Modeling and statistical methods

#### SEM

The SEM (also called Linear Structural Relations Model, Covariance Structure Modeling, Causal Modeling, and Latent Variable Modeling) approach has been developed in a general and widely accessible form during the past decade [8]. The approach was developed mainly by Jöreskog and Sörbom [9], Hayduk [10] and Bollen [11], and discussed further by Bollen and Long [12], and Marcoulides and Schumacker [13]. Historically, SEM is based on the assumption that the measured variables that generate a specific covariance structure are multivariate normally distributed, and are treated as continuous. Estimation of the structural equation model parameters is executed through normal-theory maximum likelihood (ML) methods.

The SEM methodology and analysis provide a comprehensive and flexible approach to research design and data analysis. It is a covariance matrix-based analytic tool that fosters diagrammatic framing of research questions and permits simultaneous evaluation of measurement constructs and the structural paths between those constructs. Our study of caregivers of children with CP involved a complex, comprehensive model of latent constructs affecting the well-being of caregivers. The SEM thus provides flexibility for working with multiple related equations simultaneously and gives a detailed picture of the possible causal relationships among key constructs.

The SEM was used to test specific hypotheses, as outlined in our conceptual model. Following a two-step approach recommended by Anderson and Gerbing [14], the first step involved a confirmatory factor analysis to develop an acceptable measurement model. The measurement model defined the observed variables in terms of "true" latent variables (endogenous or exogenous) and a measurement error term. At this stage, each latent variable was allowed to correlate freely with every other latent variable. In step two, the measurement model was modified to represent the postulated causal model framework. This theoretical model was then tested and revised until a theoretically meaningful and statistically acceptable model was found.

There are many different goodness-of-fit measures, reflecting different considerations, and usually three or four are reported. To assess model fit, Jaccard and Wan [15] recommended use of at least three tests which reflect diverse criteria, and Kline [2] recommends at least four. Several model diagnostic approaches were used for both the measurement and path models to assess integrity of each phase of this SEM study: the overall chi-square test, the root mean square error of approximation (RMSEA) [16], Bentler and Bonett's Non-normed Fit Index (NNFI) [17], Bentler's Comparative Fit Index (CFI) [18], and the Root Mean Square Residual (RMSR). The chi-square statistic provides a test of the null hypothesis that the theoretical model fits the data. Non-significant p-values indicate a good fit. However, the chi-square value is sensitive to sample size [19]. The chi-square test can result in the rejection of a model that appears to fit the data quite well. RMSR is often reported to show how large residuals are and as a measure of overall fit. The closer the RMSR is to 0 for a model being tested, the better the model fit. A RMSEA value close to 0.05 and NNFI or CFI values above 0.90 indicate an acceptable fit of the model. The incremental fit index NNFI that are not parsimony adjusted was suggested by Hu and Bentler [20] to 0.95 as a start of acceptable fit. This rule has been criticized as too restrictive [21]. As there exists no empirical or reasoned basis for choosing particular alternative cutoff values. Thus, 0.90 stands as the agreed-upon cutoff for overall fit indices

We conducted the SEM analysis using SAS 8.0 System's CALIS procedure [22], and the maximum likelihood estimation method was employed.

#### LLM

The LLM is an important tool for the analysis of categorical data. Its methodology was developed initially during the 1960s. Although many investigators made significant contributions, Goodman (1967) was a particularly influential researcher who popularized the method in the social sciences [23]. Bishop, Feinberg, and Holland (1975) described the methodology for the general statistical community [24].

The aim of LLM is to measure the relationships among variables in a multi-dimensional cross-tabulation. The LLM is most appropriate when there is no clear distinction between response and explanatory variables, such as when all of the variables are observed simultaneously, as in our cross-sectional sample in the Caregiver study. Log-linear models describe association and interaction patterns among a set of categorical variables, and the focus is on determining the degree of their statistical dependence. Maximum likelihood is the usual method used for estimating the parameters associated with various effects. The goodness-of-fit of the model can be tested by using both Pearson's χ^2^, which is a function of the discrepancy between the observed and estimated frequencies in the cells, or the likelihood-ratio χ^2 ^statistic (G^2^). These two statistics have asymptotically the same values, but the latter is more appropriate for the LLM [25]. The likelihood ratio test was used here to compare the fit of the independence and saturated log-linear models. The parameter estimates for the LLM indicate the effects of variables on the log of expected frequencies in the cross-tabulated cells. We performed log-linear analysis in the SAS 8.0 system by using the CATMOD procedure [22]. Text for this section.

## Results

### SEM: Measurement model

**Figure 2:**
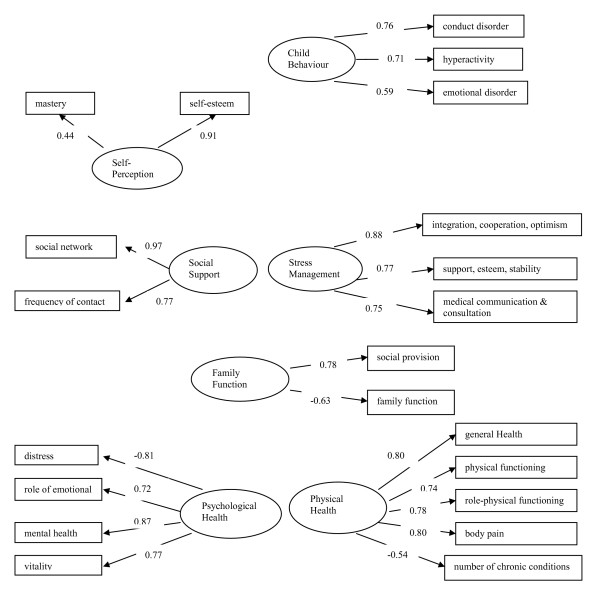
**Measurement model**: standardized factor loadings of observed variables (rectangles) on latent variables (ellipses). The loadings of 'Caregiving Demands' and 'Gross Income' were not estimated as a loading can not be estimated when there is a single indicator for a construct (not shown). Each construct (latent variables and two single indicators) is connected to every other by a curved, two-headed arrow, meaning that every construct is allowed to covary with every other construct.

Due to missing data in 18 cases, data from 450 out of 468 primary caregivers of children with cerebral palsy were analysed. Testing the fit of the measurement model led to a few refinements to the conceptual model presented in Figure 1. The final measurement model, including twenty-three observed variables, had acceptable fit to the data. The chi-square value for the final model was 544.9 with degree of freedom 197 (P-value < 0.0001). The chi-square value for the null model was 4556.5 with degree of freedom 253 (P-value < 0.0001). Technically when the proper assumptions are met the chi-statistic may be used to test the null hypothesis that the model fits the data. In practice, however the statistic is very sensitive to sample size and departure from the multivariate normality, and will very often result in the rejection of a well-fitting model. For this reason, it has recommended that the model chi-square statistic be used as a goodness of fit index, with small chi-square values (relative to the degree of freedom) indicative of a better model fit [19]. RMSEA = 0.06; RMSR = 0.05; NNFI = 0.90; and the CFI = 0.92. Nine constructs (rather than the originally hypothesized eleven) were established. Seven of them were latent variables and two were single observed variables. The standardized factor loadings of observed variables on the latent variables are presented in Figure 2. All factor loadings were substantial in magnitude, and significantly different from zero, indicating that the latent constructs were adequately operationalized by the observed variables.

### SEM: Structural model (path model)

**Figure 3:**
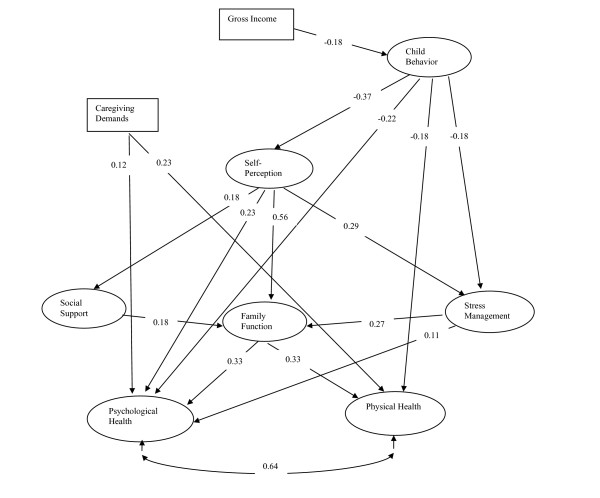
**Structural (path) model of factors influencing the health of caregivers**: standardized path coefficients appear on single-headed arrows. Correlations of the residual term of Psycho and Physical latent variables appear on curved double-headed arrows.

The path model was developed incorporating the initial hypothesized pathways and the final modified measurement model. The Lagrange Multiplier test [26] was used to suggest the addition of potentially significant paths and the deletion of non-significant ones (p > 0.05). The covariance residual (disturbance) term between Physical and Psychological Health latent variables was suggested to be correlated by the LM test. The final path model fitted the data acceptably after a few modifications. The chi-square value was 583.3 with degrees of freedom 215 (P-value < 0.0001). The RMSEA was 0.06, The RMSR was 0.059. CFI was 0.91, and NNFI was 0.90. We should be cautious about the NNFI value, as the value 0.90 was not very close to 1 and NNFI might be sensitive to the sample sizes and the distributions. All pathways were statistically significant at p < 0.05 and in the predicted direction. Figure 3 displays standardized path coefficients on the causal paths for the final path model. The standardized path coefficient (β) is the expected change (in standard deviation units of the dependent variable) that would be associated with a one standard deviation shift in the independent variable, when the other variables are held constant.

### Log-linear model

The categorical data in our log-linear model were derived from construct variables in the final measurement model, using as few levels as possible. From the confirmatory factor analysis on the Caregiver data, we had created seven latent continuous variables: Psychological Health, Physical Health, Social Support, Family Functioning, Stress Management, Self-Perception, and Child Behaviour. Caregiving Demands and SES constructs were represented by single observed variables "Caregiving Demands" and "Gross Income" respectively (Table 2). Each log-linear variable was obtained by applying a median split to its corresponding continuous variable. Categorisation of continuous variables is commonly used in clinical research. The main advantage of dichotomization is that it greatly simplifies the statistical analysis and leads to easier interpretation of results. However, dichotomization of the continuous variables split at the median should be used with caution as it can lead to a loss of information and reduced power [27]. We categorized each of these construct variables using 2 levels, with codes "0" and "1". For example, the "Child Behaviour" construct was categorized in its simplest form into "more child behaviour problems" and "fewer child behaviour problems".

**Table 2:** Descriptions of categorical variables for log-linear model

Name of constructs	Symbols of categorical variable	Description: Levels '0' and '1'
1. Psychological Health	PS	'1' for better Psychological health
2. Physical Health	PH	'1' for better physical health
3. Social Support	SS	'1' for better social support
4. Family Function	FF	'1' for better family functioning
5. Stress Management	SM	'1' for better management ability
6. Self-Perception	SP	'1' for better Self-perception ability
7. Child Behavior	CB	'1' for more behaviour problems
8. Caregiving Demands	CD	'1' for less Caergiving demand
9. Gross Household Income	GI	'1' for Higher income

Stepwise variable selection with backward elimination was used to select a parsimonious log-linear model that describes the important associations within the nine-dimensional frequency tables containing the data [28]. A summary of the LLM results is presented in Table 3.

**Table 3:** Selection of final log-linear model

Component due to	Deleted terms	G^2 ^(d.f.)	P-value
Model (1): all main effect terms		311.44(196)	<0.0001
Model (2): all two-factor interaction terms		86.13(160)	1.00
Model (3): starting model	All two-factor interaction terms related to SS	77.82(108)	0.987
Model (4): model fit well	PS*GI, GI*SM, SP*PH, GI*SP, SP*SM, GI*CB, PS*FF, FF*PH, CD*PS, GI*FF, CB*PH, GI*CD, CD*SM, CB*FF, CB*CD, CD*SP, CD*FF	101.34(126)	0.948
Difference between Model (3) and Model (4)		23.42(18)	>0.10
Model (5): final model	GI	82.38(76)	0.29

We began by choosing a significance level of 0.05, and then examined the goodness-of-fit of the following models: (1) model with main effect terms only, (2) model with all two-factor interaction terms. Since model (1) did not fit the data, and model (2) over-fitted the data (the likelihood ratio G^2 ^= 86.13, with d.f. = 160, P value = 1.00), we looked at model (2) first. We found that in model (2) none of the two-factor terms related to the Social Support variable were significant. The model without these terms (model (3)) did fit the data well (G^2 ^= 77.82 with d.f. = 108, P = 0.9872). Therefore we choose model (3) as the initial model for backward elimination, from which we deleted the non-significant two-factor terms, one at a time. Based on the corresponding changes in the G^2 ^value, we deleted non-significant two-factor terms (Table 4). Since we were unable to add or delete any more terms at this stage, we arrived at model (4). This model fit the data reasonably well, as indicated by the likelihood ratio G^2 ^test, with the value = 101.34, and d.f. = 26, P = 0.948. We observed that in model (4), there were no two-factor terms related to the "Income" variable, though its main effect was significant (P = 0.036). Excluding this single term from model (4) still provided an adequate fit to the data. The G^2 ^values for model (5) was 82.38 with d.f. 76 (P = 0.29). Therefore we took this simpler model as the final model to compare the relationships among two-factor interaction terms with the path relationships in the SEM. In Table 4 we report the estimates of the interaction parameters of this final log-linear model.

**Table 4:** Parameter estimates for the final log-linear model

Parameter (interaction term)	Unstandardized estimate	Std. error	Standardized estimate	P-value
Psycho*Physical	0.4980	0.062	8.03	<0.0001
Psycho*Stress Management	0.2205	0.0536	4.11	<0.0001
Psycho*Self-Perception	0.3034	0.0556	5.46	<0.0001
Psycho*Child Behavior	-0.1527	0.0547	-2.79	0.0052
Physical*Caregiving Demand	0.1748	0.0483	3.62	0.0003
Physical*Family Function	0.1753	0.0536	3.27	0.0011
Child Behavior*Stress Management	-0.1363	0.0521	-2.62	0.0089
Family Function* Stress Management	0.2670	0.0539	4.95	<0.0001
Child Behavior* Self-Perception	-0.2149	0.0538	-3.99	<0.0001
Self-Perception*Family Function	0.5304	0.0634	8.37	<0.0001

Parameter estimates in the LLM are analogous to effect sizes, and they may be expressed in unstandardized or standardized form. Standardized parameters are unstandardized parameter estimates divided by their standard errors and are shown in Table 4. The estimates of the two-factor interaction terms between Family Function and Self-Perception, Psychological Health and Physical Health had the largest magnitudes (standardized estimates > 8.00). A higher value of Self-Perception was associated with better Family Functioning, and better Psychological Health was positively related to better Physical Health. The lowest standardized value among the two factor interaction term was -2.62, for the interaction between Child Behaviour and Stress Management.

Effect sizes may also be expressed as odds ratios. The odds ratio is represented as exp(α), where α is the unstandardized parameter estimate. Odds ratios measure the strength of associations between variables. An odds ratio above 1 indicates a positive association between a pair of variables, while an odds ratio smaller than 1 indicates a negative association. The odds ratios are presented in Table 5 for comparison with the associations found in the path model.

**Table 5:** Comparison of the results of the SEM and the LLM approaches

	Significant test/effect measures					
Terms included in models (Terms were represented as interaction terms in LLM, but were represented as pathways in SEM except for '#')	SEM	LLM	SEM (t-values)	LLM (p-values)	SEM(Path Coefficient) (effect size*)	LLM (Odds Ratio) (effect size*)

Physical*Psycho (#)	Yes (#)	Yes	7.92	<0.0001	0.64 (#)(L)	1.65(L)
Psycho*Stress Management	Yes	Yes	2.52	<0.0001	0.11(S)	1.25(S)
Psycho*Self-Perception	Yes	Yes	3.15	<0.0001	0.23(M)	1.35(M)
Psycho*Child Behaviour	Yes	Yes	-4.07	0.0052	-0.22(M)	0.86(S)
Physical*Caregiver Demands	Yes	Yes	5.00	0.0003	0.23(M)	1.19(S)
Physical*Family Function	Yes	Yes	5.34	0.0011	0.33(M)	1.19(S)
Stress Management*Child Behaviour	Yes	Yes	-2.75	0.0089	-0.18(M)	0.87(S)
Stress Management*Family Function	Yes	Yes	4.46	<0.0001	0.27(M)	1.31(S)
Self Perception*Child Behaviour	Yes	Yes	-4.78	<0.0001	-0.37(M)	0.81(S)
Family Function*Self-Perception	Yes	Yes	6.67	<0.0001	0.56 (L)	1.70 (L)
Child behaviour*Gross Income	Yes	No	-3.36	NA	-0.18 (M)	NA
Psycho*Family Function	Yes	No	3.83	NA	0.33 (M)	NA
Physical*Child Behaviour	Yes	No	-2.98	NA	-0.18 (M)	NA
Social Support*Self-Perception	Yes	No	3.10	NA	0.18 (M)	NA
Stress Management*Self-Perception	Yes	No	4.30	NA	0.29 (M)	NA
Psycho*Caregiving Demands	Yes	No	3.12	NA	0.12 (S)	NA
Social Support*Family Function	Yes	No	3.67	NA	0.18 (M)	NA

Note: # Error Covariance between Psycho and Physical latent variables (or correlations of the Residual term of Psycho and Physical latent variables) in the SEM model; * We have ranked large, medium and small effects for 10 effect sizes in both models.

## Discussion

### 1. Comparison of results from the SEM and LLM

The SEM and LLM results were compared in terms of their similarities and differences. Table 5 compares the two sets of results in terms of significant associations and their corresponding effect sizes. The SEM results were generally supported by the LLM results with the exception that there were more significant parameters identified in the SEM results. As in Table 5, the un-bolded interaction terms were excluded from the final LLM results as they were not statistically significant.

#### 1.1. Similarities between results

The main results of the two models led to similar conclusions, and they both had reasonable fit, even though each approach started with a different conceptual framework. All ten of the effects identified as significant in the LLM results were also significant in the SEM results. The signs and effect sizes were quite consistent in the ten effects common to both models.

The strength of the associations represented by the standardized estimates of the paths in SEM was judged according to Cohen's criteria for multiple analysis of variance, i.e., large (β = 0.35), medium (β = 0.15) or small (β = 0.02) [29]. The effect size represented by the unstandardized parameter estimate in LLM was also determined by Cohen's criterion for the Chi-squared test for goodness of fit or association in contingency tables: large (α = 0.50), medium (α = 0.30) and small (α = 0.10) [25]. Equivalently, an odds ratio of 1.65 (or 0.61 for negative association), represents a large effect for a positive association, an odds ratio of 1.35 (or 0.74) represents a medium-sized size effect, and an odds ratio of 1.15 (or 0.87) represents a small effect.

There was a large effect of Self-Perception ability on Family Functioning in the SEM and also a large odds ratio between these two factors in the LLM. These two terms had the largest effect size in both models. Another large effect size in the LLM was the interaction term of Psychological Health and Physical Health. The relationships of the two health-related latent variables may be considered bi-directional in the SEM analysis. We did not hypothesize bi-directional relationships between these two latent variables in the conceptual model, because including bi-directional effect often results in an unstable SEM and with inadequate overall fit. However, the covariance residual between these two latent variables had a significant effect, and its effect size was large as well. This implied that Physical Health and the Psychological Health were indeed highly related.

In the SEM, higher levels of Self-Perception were significantly associated with better caregiver Psychological Health. This association was consistent with the corresponding moderate relationships observed in the LLM results. Better Stress Management strategies were associated with better caregiver Psychological Health in the SEM and also in the LLM. The effect sizes were both small and in the same direction.

The following six associations were similar in both the SEM and the LLM: having more Child Behaviour problems was associated with lower quality of caregiver Self-Perception and use of fewer Stress Management strategies; better Stress Management was associated with better Family Functioning; better Family Functioning was associated with better caregiver Physical Health; more Child Behaviour problems were negatively associated with Psychological Health; decrease of Caregiving Demands was associated with better caregiver Physical Health. All these terms in the SEM had moderate effects and also showed small effects in the LLM and were always in the same direction.

#### 1.2. Differences between results

We noted that there were seven additional significant pathways in the SEM that were not significant in the LLM (Table 5). These effect sizes (absolute values) ranged from 0.12 to 0.33, so none of them was large.

The Social Support factor was dropped from the LLM, suggesting that this factor was independent of the other factors. However in the SEM there were two pathways which were related to this construct: better Self-Perception was positively associated with Social Support, and better Social Support was associated with better Family Functioning.

Gross Income was the only single significant term in the LLM, and there were no other two-interaction terms involving this factor; excluding it still provided an adequate fit to the data. Therefore we took the simpler model (without the Gross Income term) as our final model for comparing with the SEM. However, in the SEM there was one direct pathway from Gross Income to Child Behaviour construct, though its effect size was not very large (0.18).

The other four significant pathways (i.e., Caregiving Demands to Psychological Health, Child Behaviours to Physical Health, Self-Perception to Stress Management and Family Functioning to Psychological Health) in the SEM were not significant in the LLM approach.

#### 1.3. Conclusions

**Figure 4:**
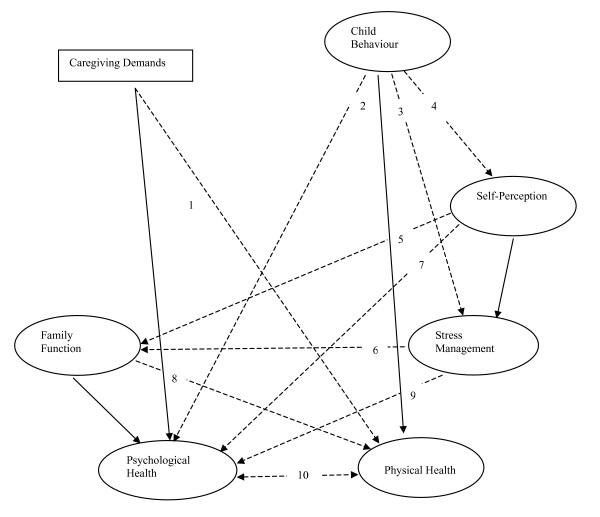
**Diagram of main results from two methods**: dashed lines with arrow represents for similar relationships found in both the log-linear model and structural model. Solid lines with arrow represented for pathways in the structural model only.

Figure 4 summarizes the results from the two different methods. In a head-to-head comparison, we noted that most of the important findings were consistent in the two models.

Child Behaviour affected the Psychological Health of caregivers (dashed line 2 in Figure 4) in both the SEM and LLM approaches. Child Behaviour problems were the most important predictors of caregiver psychological wellbeing. Also, Self-Perception and Stress Management were associated with the Psychological Health of caregivers (dashed line 7 and 9 in Figure 4) in both models. They played an important role in mediating the negative impact of stressors on caregivers. The SEM results also indicated that Caregiving Demands and Family Function influenced Psychological Health of caregivers, but there was no evidence of these relationships from the log-linear model.

Caregiving Demands and Family Functioning were important factors directly related to the Physical Health of caregivers in both models (dashed lines 1 and 8 in Figure 4). Child Behaviour influenced Physical Health of caregivers from the SEM model. However, the association between Child Behaviour and Caregiver Physical Health was not statistically important in the log-linear model.

It was clear from both analyses that Family Functioning, Self-Perception and Stress Management were very important mediator factors. Self-Perception with Family Functioning (dashed line 5 in Figure 4) and Stress Management with Family Functioning (dashed line 6 in Figure 4) were associated significantly in both models, and these coping factors played important roles in the caregiving process.

Furthermore, Child Behaviour was associated with Self-Perception and Stress Management in both models (dashed line 3 and 4 in Figure 4). The significant association between Self-perception and Stress Management appeared to be important in only the SEM.

We should also specify that Psychological Health and Physical Health were highly correlated in both models (dashed double-headed line 10 in Figure 4). However, in the SEM the causal relationship between the Psychological and Physical Health outcome variables was not tested, but the covariance residual between these two latent variables had a significant effect.

### 2. Comparison of overall structures

The Structural Equation Model was a representation of theoretically derived causal relationships between hypothesized constructs, and not merely the simple associations between those constructs. SEM also allows insight into direct and indirect pathways, while log-linear models do not. But higher-level interaction terms in the LLM might demonstrate confounding and interaction structures that might relate to direct pathways in the structural model. We considered only two-factor interaction terms in this LLM analyses, as this led to a more parsimonious model (models including three-factor interaction or higher level interaction terms over-fitted the data in our case). Overall, the results from the SEM and LLM approaches were comparable; the most important significant causal relationships among those caregiver relevant factors in the path model corresponded to significant associations between the same factors in the LLM.

Log-linear models require discrete measurements, and from dichotomizations of the continuous variables, some statistical information is lost; on the other hand there is more robustness in LLM because less parametric modeling is assumed than with the SEM. Thus the results in the SEM incorporated more detailed information from the data than did the log-linear model. On the other hand, the LLM approaches led to a more parsimonious model due to the fewer levels of the factors as well as the strategy in reducing unimportant variables and identify higher order interaction effects. The advantage of this method is therefore that it may converge more easily and provide more parsimonious results than those obtained from the SEM analysis. The LLM is a more data-driven approach, and it can be seen as a potential validation of the SEM findings, or as a kind of sensitivity analysis.

### 3. Comparisons of model specification and testing

The strategy of SEM development is complex and can have low stability compared with the simpler form of the LLM. Indeed, SEM is usually viewed as a confirmatory rather than exploratory procedure, using one of three approaches [30]:

(1). Strictly confirmatory approach: A model is tested using SEM goodness-of-fit tests to determine if the pattern of variances and covariances in the data is consistent with a structural model specified by the researcher. However, as other unexamined models may fit the data as well or better, an accepted model may be merely one that has not yet been invalidated.

(2). Alternative model approach: One may test two or more competing causal models to determine which has the best fit. But in most specific research topic areas, the researcher rarely has the luxury of two such models being available.

(3). Model development approach: In practice, much SEM research combines confirmatory and exploratory purposes: a model is tested using SEM procedures, is found to be deficient, and an alternative model is then tested based on changes suggested by SEM modification indexes such as the Lagrange multiplier index, the Wald test index, etc. This is the most common approach found in the literature and it was employed in our case.

One of the major practical problems in a model development approach is that the methods are very demanding of researchers' methodological skills and content knowledge of the application. Model fit itself is subjective, because there is no single 'correct' model and other models may be equally plausible given the same sample. The SEM involves testing a theoretically derived model; any changes to the theoretical model based on data-driven considerations may result in a model incorporating concepts that do not match well with the originally intended model [10].

In our SEM study, the final measurement model was somewhat different from the initially hypothesized one, because of the findings in the preliminary analysis and because of some refinements to the conceptual model that were made following the confirmatory factor analysis. Refinements of the measurement model also led to changes in our path model. In contrast, because the log-linear modeling method is a more data driven approach, it converges more easily and is relatively robust.

When utilizing structural modeling methods, one will frequently find some practical problems emanating from the statistical results. These problems may make the results difficult to interpret or even misleading. Hence testing and improving SEM models requires care. One of the common problems in fitting the model is that variances may sometimes be estimated as negative or zero (the so-called "Heywood case") [31]. Such estimates are not only meaningless, but also inappropriate since negative variances are inadmissible. One accepted solution is to reparameterize the model to guarantee non-negativity [32]. In the initial measurement model, SES was hypothesised to be measured by Gross Income and Education indicators. During our testing of the measurement model, we found there was a negative residual variance for the "Income" variable. To solve this problem, we re-ran the model with this variance set to zero, and then evaluated whether the zero variance estimate was compatible with the conceptual design of the study. Fortunately, our results were essentially unchanged. A possible interpretation for this is that the residual variance is not significantly different from zero, and so we assume that "Income" summarizes the entire SES construct. This implied that "Education" was not required for the SES construct, and could be removed.

## Conclusion

In summary, we found no important differences in the substantive conclusions of the two models. Although consistent results were found using these two approaches, the inconsistent findings may exist due either to differences in their conceptual frameworks, or to differences in their statistical approaches. The broad confirmation of the SEM results by the LLM provided some reassurance that the SEM had been adequately specified, and that it broadly fitted the data. The log-linear analysis provided a summary of the important results, but based on a simpler model specification, and based on less data information content from the discretized variables. Although one can never be certain in such matters, the similarity of results between the two approaches suggests that the results of the SEM are valid.

## Competing interests

The author(s) declare that they have no competing interests.

## Authors' contributions

SDW initiated the idea and contributed the crucial input to the paper. BZ wrote the initial draft, and all authors contributed to the subsequent drafts. All have read and approve of the final draft.

## Pre-publication history

The pre-publication history for this paper can be accessed here:


